# Supplementary material for: THz irradiation inhibits cell division by affecting actin dynamics
Source: PLoS One. 2021 Aug 2;16(8):e0248381. doi: 10.1371/journal.pone.0248381 (PMC8328307; doi:10.1371/journal.pone.0248381)
Supplement: S1 File — (DOCX) [file pone.0248381.s001.docx]

Supplementary Materials for

**THz irradiation inhibits cell division by affecting actin dynamics**

Shota Yamazaki^*^, Yuya Ueno, Ryosuke Hosoki, Takanori Saito, Toshitaka Idehara, Yuusuke Yamaguchi, Chiko Otani, Yuichi Ogawa, Masahiko Harata^*^, Hiromichi Hoshina^*^

*Corresponding author. Email: shota.yamazaki.fc@riken.jp, masahiko.harata.b6@tohoku.ac.jp, hoshina@riken.jp

**This PDF file includes:**

Supplementary Text

Figs. S1 to S3

Movies S1

**Other Supplementary Materials for this manuscript include the following:**

Movies S1

**Temperature change of the sample due to THz irradiation**

Here, we estimate the temperature change of the sample subjected to pulsed THz irradiation with a frequency of 0.46 THz, a pulse energy of 5.7 mJ/cm^2^, a pulse duration of 10 ms, and a repetition rate of 1 Hz. The peak power density of the radiation (gyrotron) is 0.6 W/cm^2^.


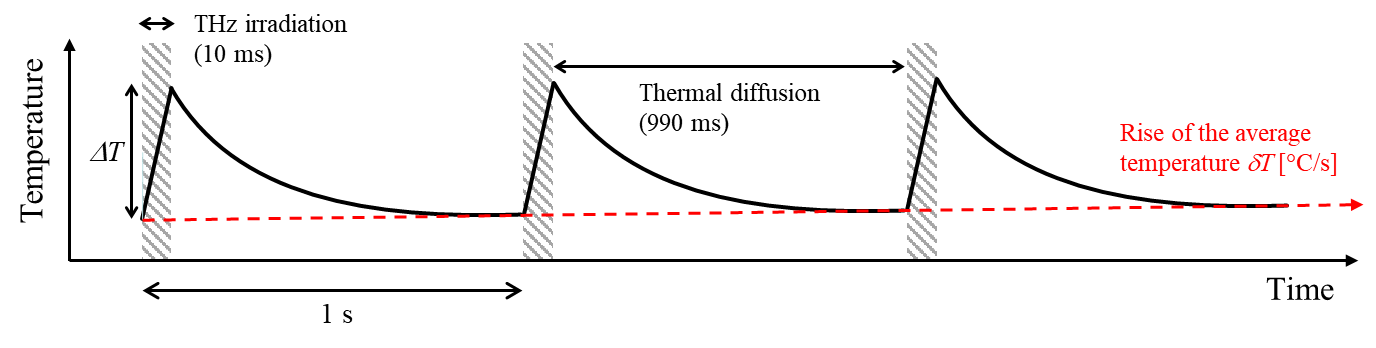


**Figure S1.1 Temperature change of the sample during THz irradiation.**

Figure S1.1 describes the temperature profile of the sample during THz irradiation. When the THz pulse arrives, water absorbs most of the energy and the temperature of the sample increases adiabatically in 10 ms (*ΔT*). After the pulse has passed through the sample, the temperature decreases owing to thermal diffusion until the next pulse arrives. In total, the temperature of the sample increases for *δT* in 1 s. The temperature of the sample was kept at 37 °C by the PID program of the heating stage. In this section, *ΔT* and *δT* are estimated using an adiabatic model and the finite-element method, respectively.

Figure S1.2 shows a schematic representation of the sample with a thickness of *dx* and surface area of *S,* irradiated by a THz pulse with a pulse energy of *I* [J].


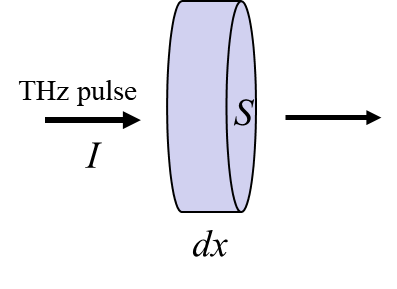


**Figure S1.2 Model of THz irradiation of the sample.**

According to the Beer–Lambert law, the absorbed THz energy, *ΔI* [J], is written as

$\Delta I=-I(log10)\alpha dx$ (1)

where *α* [cm^−1^] is the absorption coefficient of the sample. The temperature increase, *ΔT*, [°C] is

$\Delta T=\frac{\Delta I}{\rho Sdx}$ (2)

where *ρ* [J/°C⋅cm^3^] is the specific heat of the liquid water. Introducing the pulse energy density, *P*, [J/cm^2^] by the relation $I=P\cdot S$, *ΔT* can be written as follows:

$\Delta T=\frac{log10*P[\text{J/c}\text{m}^{\text{2}}]\alpha[\text{c}\text{m}^{\text{-1}}]}{\rho[J/C\mathrm{cm}^{3}]}$ (3)

In this study, the absorption coefficient of liquid water (74 cm^−1^) was used as *α*. Using a value of *ρ* = 4.2 J/°C⋅cm^3^, *ΔT* was estimated as 0.23 °C. The temperature exponentially decayed in the sample after irradiation, as shown in Fig. S1.3.

**Figure S1.3 *ΔT* after the irradiation with a THz pulse**

After THz irradiation, the temperature changed because of thermal diffusion. The time-evolved temperature distribution was simulated using the commercial finite-element method software (COMSOL Multiphysics®). Figure S1.4 shows the geometry of the simulation model. The THz beam is vertically irradiated from the top through the air and the polyethylene film (which represents the film dish) and heats the surface of the water. To simplify the model, only the cylindrical area with a radius of 4 mm and thickness of 0.1 mm was heated by 0.23 K at *t*= 0.

The thermal distribution was simulated for 1 s in 10-ms steps. Figure S1.5 shows the simulated cross-section of the thermal distribution at *t* = 0, 200, and 1000 ms. The temperature at the surface of the sample drops to 300.03 K at *t*= 1000 ms.


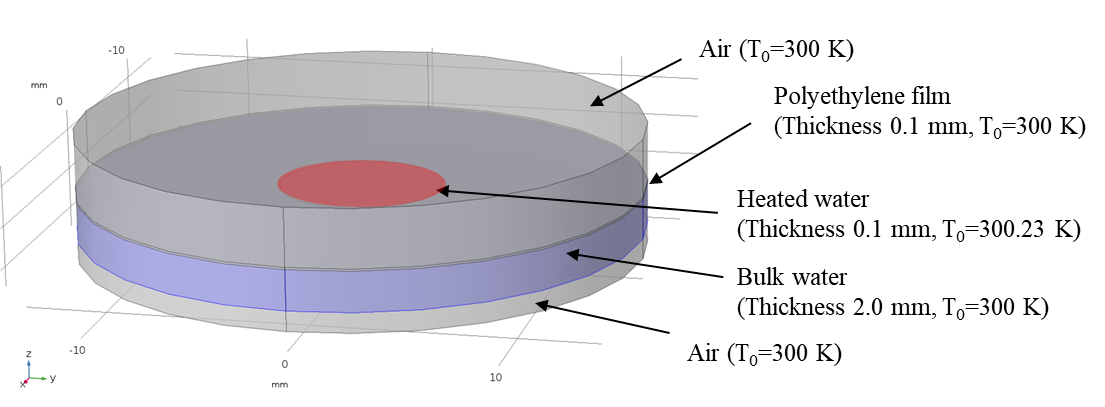


**Figure S1.4 Geometry of the simulation model.**


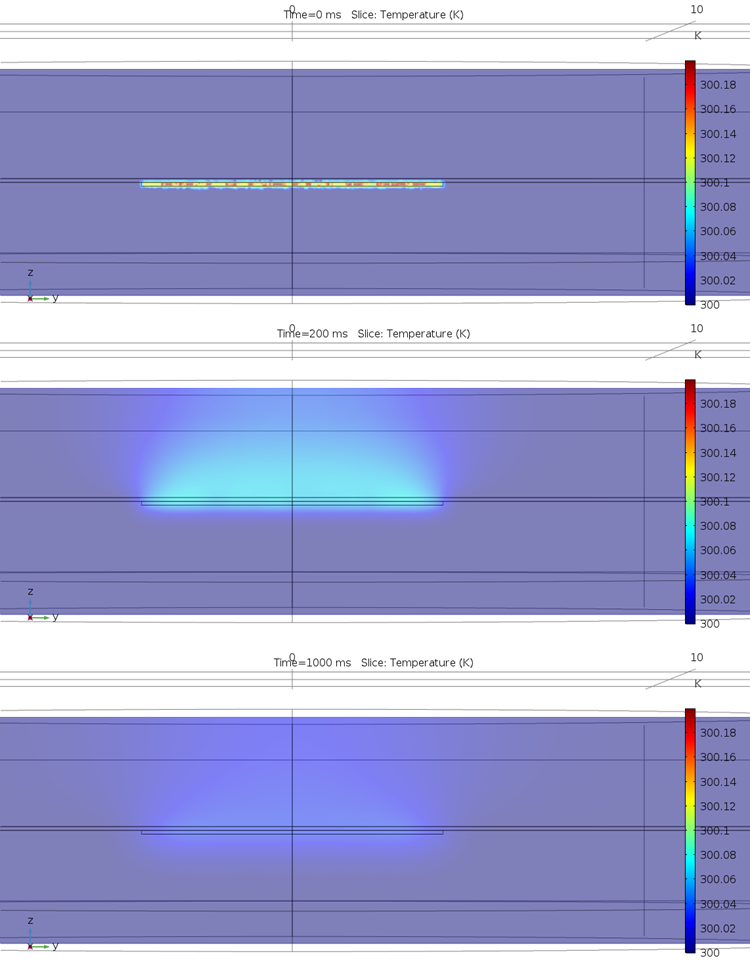


**Figure S1.5 Simulated cross-section of the thermal distribution at *t* = 0, 200, and 1000 ms.**

In summary, the temperature change during THz irradiation, *ΔT*, was estimated as 0.23 °C using the adiabatic model. The temperature rise after thermal diffusion, *δT*, was estimated as 0.03 °C using the finite-element method. Even when the effect of *δT* was integrated for 10 s, the temperature change of the sample was only 0.3 °C, which was easily controlled by the heating stage. Therefore, we conclude that the sample temperature rises at most by 0.23 °C as a result of irradiation.


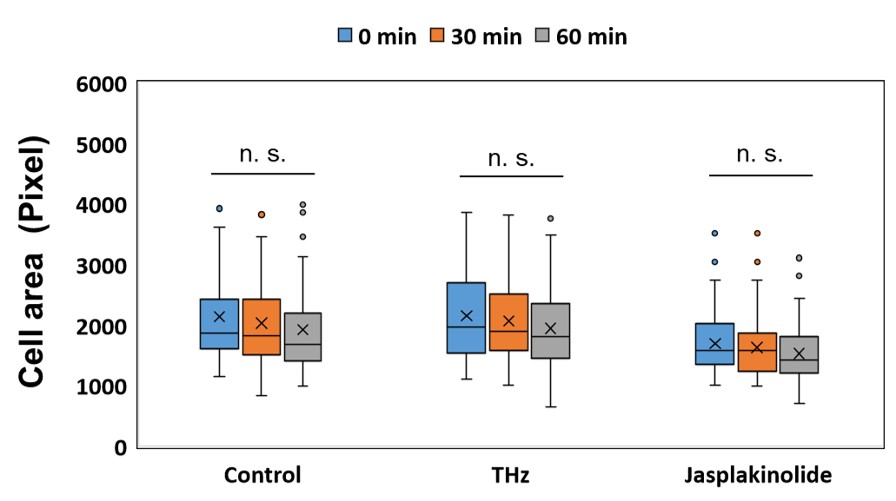


**Fig. S2.** **Morphological analysis of cells.**

The outlines of cells were selected using the area selection tools in the Fiji software, and the cell areas and perimeters were measured. Cell areas were measured from the microscopy images at 0, 30, and 60 minutes. The box plot shows the mean values and the standard deviation of three independent experiments. More than 52 cells were measured in each experiment.


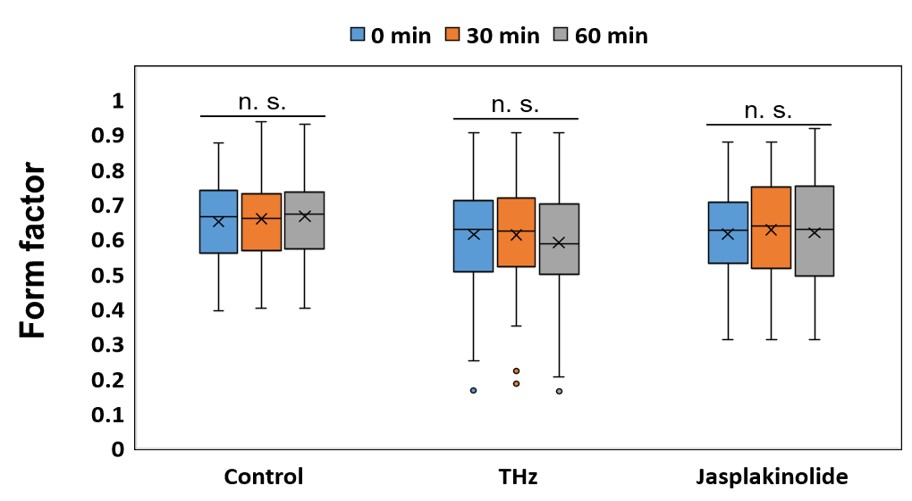


**Fig. S3.** **Morphological analysis of cells.**

The cell shape was determined from the form factor. The form factors of individual cells were calculated as 4*πS*/*L*^2^, where *S* is the projected cell area and *L* is the cell perimeter. The box plot shows the mean values and the standard deviations of three independent experiments. More than 52 cells were measured in each experiment.
